# Provision of antiretroviral treatment in conflict settings: the experience of Médecins Sans Frontières

**DOI:** 10.1186/1752-1505-4-12

**Published:** 2010-06-17

**Authors:** Daniel P O'Brien, Sarah Venis, Jane Greig, Leslie Shanks, Tom Ellman, Kalpana Sabapathy, Lisa Frigati, Clair Mills

**Affiliations:** 1 Public Health Department, Médecins Sans Frontières, Amsterdam, Netherlands; 2 Department of Infectious Diseases, Geelong Hospital, Geelong, Australia; 3 Victorian Infectious Diseases Service, Royal Melbourne Hospital, Melbourne, Australia; 4 Manson Unit, Médecins Sans Frontières, London, UK; 5 School of Child and Adolescent Health, Red Cross Childrens' Hospital, Capetown, South Africa; 6 Te Kupenga Hauora Maori, Faculty of Medical and Health Sciences,University of Auckland, Auckland, New Zealand

## Abstract

**Introduction:**

Many countries ravaged by conflict have substantial morbidity and mortality attributed to HIV/AIDS yet HIV treatment is uncommonly available. Universal access to HIV care cannot be achieved unless the needs of populations in conflict-affected areas are addressed.

**Methods:**

From 2003 Médecins Sans Frontières introduced HIV care, including antiretroviral therapy, into 24 programmes in conflict or post-conflict settings, mainly in sub-Saharan Africa. HIV care and treatment activities were usually integrated within other medical activities. Project data collected in the Fuchia software system were analysed and outcomes compared with ART-LINC data. Programme reports and other relevant documents and interviews with local and headquarters staff were used to develop lessons learned.

**Results:**

In the 22 programmes where ART was initiated, more than 10,500 people were diagnosed with HIV and received medical care, and 4555 commenced antiretroviral therapy, including 348 children. Complete data were available for adults in 20 programmes (n = 4145). At analysis, 2645 (64%) remained on ART, 422 (10%) had died, 466 (11%) lost to follow-up, 417 (10%) transferred to another programme, and 195 (5%) had an unclear outcome. Median 12-month mortality and loss to follow-up were 9% and 11% respectively, and median 6-month CD4 gain was 129 cells/mm ^3^.

Patient outcomes on treatment were comparable to those in stable resource-limited settings, and individuals and communities obtained significant benefits from access to HIV treatment. Programme disruption through instability was uncommon with only one program experiencing interruption to services, and programs were adapted to allow for disruption and population movements. Integration of HIV activities strengthened other health activities contributing to health benefits for all victims of conflict and increasing the potential sustainability for implemented activities.

**Conclusions:**

With commitment, simplified treatment and monitoring, and adaptations for potential instability, HIV treatment can be feasibly and effectively provided in conflict or post-conflict settings.

## Introduction

Many countries ravaged by current or recent conflict have substantial morbidity and mortality attributed to HIV/AIDS [1]. Sub-Saharan Africa, with the world's highest burden of conflict, is home to around 70% of HIV-infected people and has the largest unmet need for antiretroviral treatment (ART), estimated at around 5 million[2,3]. One analysis of HIV treatment in conflict-affected regions of northern Uganda found access extremely limited, particularly in remote and rural areas [4].

Conflict carries with it factors that can worsen the severity and progression of HIV disease such as food insecurity, contaminated water supplies, physical and psychological stress, and higher rates of other infectious diseases. Furthermore, while there is conflicting evidence on the issue [1], post-conflict, and to a lesser extent, conflict situations may increase susceptibility to HIV transmission [5]. ART can both significantly reduce HIV related mortality and morbidity [6] and potentially reduce HIV transmission [7]. However, despite increasing access to life-saving ART in more stable environments, these treatments have been uncommonly available in conflict or post-conflict settings [5,8].

Reasons for the relative neglect of conflict settings include: limited access to affected populations; poor health infrastructure and resources; a lack of prioritization of HIV-related health needs given limited resources and competing medical priorities; recommendations against providing ART in current international guidelines for health care in these environments[9,10]; a fear of the complexity of HIV treatment and lack of relevant guidelines and examples to follow; the unstable nature of the situation leading to concerns about interrupting treatment leading to antiviral resistance; and a belief that unless the provision of ART can be maintained lifelong then ART should not be initiated[8]. Nevertheless, it has been demonstrated that HIV treatment in conflict settings is both feasible and effective [11-14] and guidelines have been produced[15].

The international aim of universal access to HIV care [16] cannot be achieved unless the needs of populations in conflict-affected areas are addressed. As treatment access increases, ever larger numbers of people on ART in currently stable areas are at risk of treatment disruption if conflict affects health services or forces their migration. Therefore there has been a call to increase access to HIV treatment and prevention in these settings [8,17].

**Figure 1:**
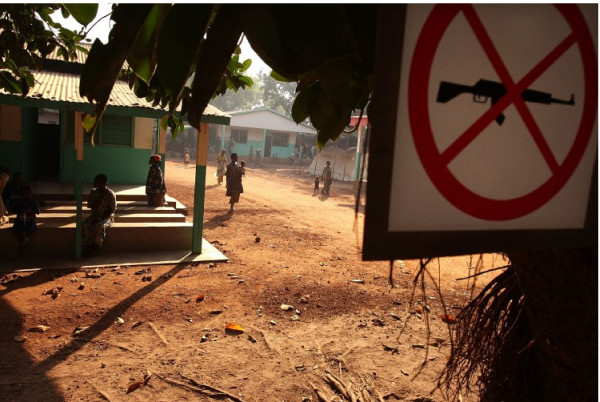
**An MSF clinic in Kabo, Central African Republic, 2007; with a sign indicating "no guns allowed in the clinic"**. Copyright Spencer Platt/Getty Images.

**Figure 2:**
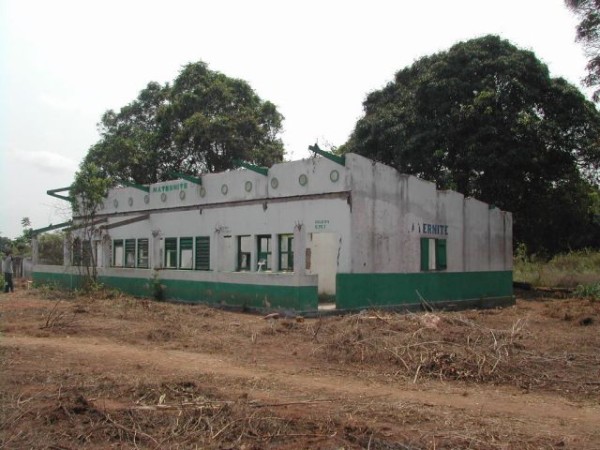
**Loulambo, Pool region, Republic of Congo, 2003; a maternity ward destroyed by conflict**. Copyright Patrick Deschamps/MSF.

**Figure 3:**
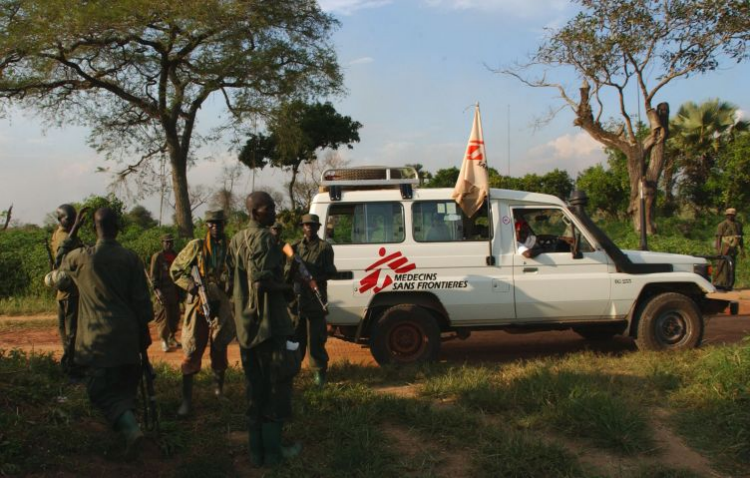
**An MSF car in conflict-affected northern Uganda**. Copyright Keith Philip Lepor.

Médecins Sans Frontières (MSF) is a humanitarian organization providing medical care to populations in crisis. MSF works in many conflict-affected settings responding to the acute health needs of affected populations amidst the breakdown of health services (Figures 1, 2, 3). In many of these programmes, urgent and considerable HIV-related health needs have been identified. Since provision of ART could significantly reduce mortality and morbidity, the MSF operational section of Amsterdam (MSF-OCA) began to introduce HIV care and treatment activities into these programmes.

**Figure 4:**
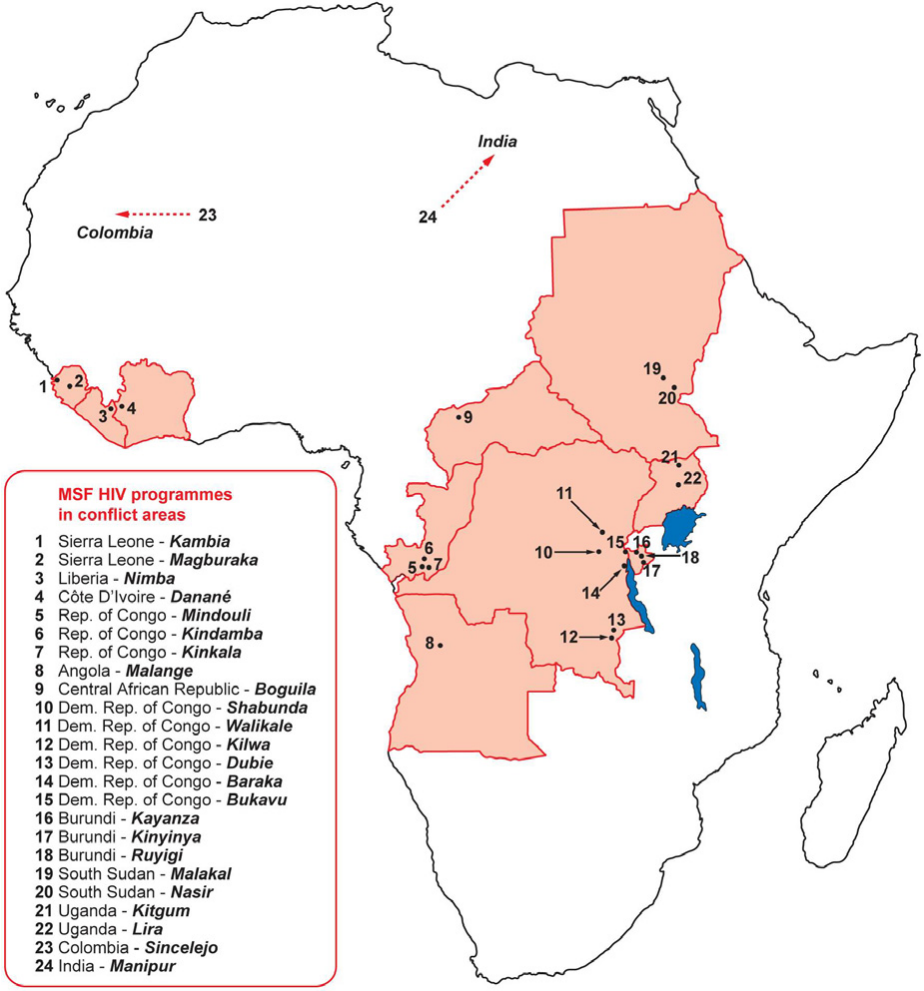
**Map showing programme sites in Africa**.* Sites in Manipur, India; and Sincelejo, Colombia not included.

An HIV programme was initiated in Bukavu, a conflict-affected region of eastern Democratic Republic of Congo (DRC) in October 2003 and expanded to 24 basic health-care programmes in 12 countries (Figure 4). We describe the contexts, activities, outcomes, challenges, and lessons learned from these programmes, including answers to common issues raised about potential difficulties in implementing such programmes. Our aim is to share the knowledge and experience obtained by MSF and its counterparts and to facilitate and advocate increased commitment for provision of HIV treatment in these settings.

## Methods

### Programme contexts

We define a conflict setting as one with active intrastate or interstate conflict. A post-conflict setting is defined as one within 2 years of a peace agreement being signed and adhered to between warring parties where often there has been only a minimal return of populations and functioning of health services due to ongoing or potential instability.

Additional file 1, Table S1 shows programme contexts and settings. 22 programmes were in sub-Saharan Africa. Thirteen were classed as conflict and eleven as post-conflict. Most (21) were in rural locations including two in refugee camps. Most countries had endured decades of instability, with conflicts lasting an average of 18 years; MSF had typically been present for 17 years. Additional file 1, Table S1 shows estimated WHO/UNAIDS country HIV prevalence data for 2005. Most countries were in Central and West Africa where prevalence is low to medium (1-10%) compared with higher rates in more stable southern Africa (10-50%) [18]. However, these rates usually reflect the situation in urban and stable areas where testing has been performed. In many of the areas where our HIV programmes were introduced there had been no prior HIV testing. ART coverage rates varied from 1% in Sudan to 51% in Uganda [see Additional file 1, Table S1] [19], but considerable in-country variation exists with rates as low as 2-122 per 1000 of eligible individuals receiving ART in conflict-affected regions of northern Uganda [4].

### Design of the HIV programmes

The aim was to treat patients attending health facilities supported by MSF, rather than to comprehensively address the HIV epidemic in programme areas. Therefore, programmes focused on patients presenting to the health facilities with an increased likelihood of having HIV or where knowledge of HIV status affected medical care. This included medical inpatients, children in therapeutic feeding centres not responding to treatment, pregnant women, and patients with tuberculosis, sexually transmitted infections, or illnesses suggestive of HIV such as severe candidiasis [see Additional file 2, Table S2]. Here we describe aspects of the programmes focusing on those important in conflict settings.

#### Integration of programmes

HIV services were integrated as much as possible into existing health facilities and activities, apart from Bukavu in DRC and Malange in Angola which, being the earliest conflict affected programmes, commenced when MSF HIV programming had a purely vertical approach. Most programmes attempted to work with national AIDS programmes, recognising the eventual need to handover patients. This ranged from discussions and agreements with Ministry of Health (MoH) at national level, to full integration with MoH staff and protocols in clinics and use of MoH drug supplies.

#### Diagnostic and treatment protocols

HIV testing was initially offered using the client-initiated model, but with the focus on providing care to those in medical need, this approach shifted from 2005 to a provider-initiated testing model[20]. Two parallel HIV rapid diagnostic tests (usually Determine HIV-1/2^® ^and UniGold HIV^® ^) were used; to facilitate integration with MoH, occasionally serial testing was implemented (eg Burundi). Confirmation testing with Orgenics Immunocomb Combfirm^® ^HIV test was introduced from 2006[21]. For ART, generic drugs were used in fixed-dose combinations. Eligibility criteria for ART and first-line regimens were standardised and based on WHO recommendations.[22]

#### Human resources

To initiate HIV activities additional human resources were usually introduced - clinical, counselling, or laboratory staff - often supported by temporary expatriate staff with HIV experience to set up systems and training [12]. The HIV component was only part of an individual's workload. Doctors caring for patients with HIV would also work in general inpatient wards, maternity, and tuberculosis services; HIV counsellors often worked in psychosocial counselling; and nurses on general medical wards. Clinical consultations were performed by doctors or nurses, though an emphasis was placed on task shifting with nurses often involved in ART care, and community health workers or patients involved in HIV counselling and adherence training.

#### Monitoring and data collection

Monitoring was clinical, supported in most projects by a limited range of laboratory investigations such as CD4 counts and liver function tests [see Additional file 1, Table S1]. Often programmes began with clinical monitoring, and laboratory investigations were introduced later. Data collection and programme monitoring usually utilized standardised FUCHIA software (Epicentre, Paris). Data were collected at each consultation using standardised forms and entered centrally into an electronic database by dedicated clinicians or clerical staff. Enrolments, survival, defaulter rates, tuberculosis rates and treatment, and immunological status on ART were regularly monitored using standardised MSF HIV indicators.

#### Sexual violence

All programmes had a gender-based violence component, including treatment for sexually-transmitted infections, emergency contraception, counselling, and access to HIV post-exposure prophylaxis (PEP). Although knowledge of HIV status is desirable, to avoid further stress to rape victims and delays, post-exposure prophylaxis was generally given before HIV counselling and testing, which was organized within the subsequent few days. The rationale was that a few days of antiretroviral drugs for an HIV-positive person would not promote anti-viral resistance, especially as non-nucleoside reverse transcriptase inhibitor (NNRTI)-based regimens were not used. Most victims presented for care after the minimum time for PEP (<72 hours), often months to years after the event.

#### Contingency planning

Contingency plans covered a variety of scenarios, from complete long-term evacuation of the programme, to short-term partial breaks in care delivery. Staff and patients were prepared for evacuation of staff, reduction of medical activities, limitations of movement, breakdown of usual communication systems, and looting. Patients were prepared to cope with forced displacements, limitation of movement, and rupture of personal medical stocks. Detailed descriptions of contingency planning have been reported [11,12,23,24] and are summarised and updated in appendix 1.

### Data analysis

Data were analysed using automated reports available in FUCHIA software (v1.6.2.526) through the R programme, which provide patient outcomes by month or by time in ART cohorts. Detailed patient data were exported from FUCHIA according to time on ART cohorts and analysed further using Microsoft^® ^Office Excel^® ^2007 and STATA 10.0 (StataCorp, Texas).

## Results

### Programme outcomes

Additional file 2, Tables S2 and S3 show programme data and outcomes. More than 10 500 people were diagnosed HIV-positive and received medical care. Median overall HIV prevalence in tested patients was 12% (range 2-45%) and was as high as 78% in tuberculosis patients (median 13%, range 4-78%). A comparison of MSF antenatal clinic data with WHO/UNAIDS 2005 national HIV prevalence estimates[18] showed that HIV prevalence was generally lower than expected - e.g. Shabunda, DRC (1% *vs *2-5%), Boguila, Central African Republic (1% *vs *6-7%), and Danane, Cote D'Ivoire (3% *vs *5-10%). This may result from limited population movement and mixing in conflict-affected regions and is consistent with research that suggests conflict may limit HIV transmission [1].

In the 22 programmes where ART was initiated, 4555 (43%) HIV-positive individuals started ART, including 348 children ( < 15 years). Follow-up information was incomplete in two programmes, and the numbers of children on ART were too small to make robust conclusions. Therefore, analyses were restricted to adults in the remaining 20 programmes (n = 4145). Of these, by the time of analysis, 2645 (64%) remained on ART in the programme, 422 (10%) had died, 466 (11%) lost to follow-up, 417 (10%) transferred to another programme, and 195 (5%) had an unclear outcome [see Additional file 2, Table S2].

More detailed further analysis was performed for adults in 12 programmes [see Additional file 2, Table S3], limited by not having complete data for all programmes due to either loss of Fuchia data post-closure (2 projects) or because Fuchia was not implemented (8 projects). ART baseline data revealed patients that were young (median age 35 years), predominately female (median 66%), severely immunosuppressed (median proportion WHO stage 3/4 80% and median baseline CD4 139 cells/mm ^3^), and ART-naïve (median 94%). For the 2572 (61%) adults with 12-month data, median 12-month survival was 0.89 (95% CI 0.88-0.91) and proportion lost to follow-up was 0.11 (95% CI 0.09-0.12) [see Additional file 2, Table S3]. In addition, robust immunological gains were achieved in our cohorts with median 6-month CD4 gain of 129 cells/mm ^3^. Median follow-up time on ART was 11.8 months (IQR 3.9-22.7).

## Discussion and Evaluation

Many obstacles have contributed to the lack of ART programs in conflict or post-conflict affected areas. They include issues related to health need prioritisation, feasibility, effectiveness, safety and ethics. Our study provides important information that can be useful in addressing many of these concerns.

### 1. "HIV treatment is complex. It will not be possible to assure safe and effective treatment in conflict affected settings."

Experience from vertical HIV programmes in resource-limited settings has led to simplification of treatment with standardised treatment protocols, fixed-dose combination drug regimens, minimal monitoring, and intensive adherence support. This approach has proved safe and effective [25] and can be applied without major changes in conflict settings. In our programmes, fixed-dose combinations facilitated adherence, procurement, and stock management, and reduced costs. Relatively complex monitoring tools such as CD4 counts and liver function tests allowed a paradoxical simplification of management and increased the ease of decision making by less experienced clinical staff [12]. HIV activities were introduced in a stepwise manner that avoided overwhelming teams and allowed staff time to gain experience in HIV care and systems to be put in place.

The evidence presented here and previously from our programmes and from others suggests that ART outcomes are equivalent to those in stable resource-limited settings [11-14]. In this study, the median 12-month survival of 0.89 (0.88-0.91) compares favourably with that in Malawi (0.81;0.79-0.83),[26] Zambia (0.82) [27] and South Africa (0.93;0.92-0.94) [28]. In addition, it is comparable with the ART-LINC study, to date the largest combined analysis of cohorts in stable resource-limited settings;[29] mortality in our programmes (9% versus ART-LINC 6%) and lost-to follow-up rates (11% versus ART-LINC 15%) after 12 months of treatment, as well as median immunological gains after 6 months of treatment (129 cells/mm ^3 ^versus ART-LINC 106 cells/mm ^3^) were similar. This analysis involves a very large dataset involving many programmes in conflict affected environments and thus provides important data supporting the effectiveness of providing ART in these environments.

### 2. "Adherence to treatment is likely to be poor due to forced displacement and population mobility."

There is no evidence that, providing the drug supply is well-managed, people will be any less adherent to ART in conflict settings. Our experience is that people adhere well [11], but treatment should be provided free of charge given the negative impact of user fees on access to services and adherence to ART [30]. Acute programme disruptions were uncommon and rarer than expected; the only programme to face such disruption was Bukavu, DRC, for 2 weeks in 2005[11]. Disruptions can occur even in 'stable' settings, either due to unexpected conflict such as in Kenya in 2008[31] or through drug ruptures secondary to mismanagement or financial limitations [32]. Thus many of the practical measures used in these settings could be applied in all HIV programmes and also to particular populations at higher risk of interruption such as migrants and nomadic populations.

With the proliferation of access to treatment in stable settings, leakage of drugs into the informal sector is inevitable even in conflict settings, and the absence of treatment programmes forces people to look to these costly and sub-standard sources. In such contexts provision of treatment is also a form of harm reduction.

Populations in conflict and post-conflict settings can be mobile. In Liberia, our patients would often cross the borders into neighbouring Sierra Leone and Guinea seeking health care or better conditions. Programmes need to allow for population movement (Appendix 1). If population movements are planned in advance, management strategies can include the provision of 3-6 months of ARVs for patients stable on treatment, or if medically stable, delaying initiation of ART until the patient arrives in the new destination [15]. Simple, cheap, and readily available regimens may be preferred to more complex and expensive ones that may not be available elsewhere.

### 3. "Treatment should be life-long. People may be started on treatment, only to stop after 6 months or a year."

In resource-limited settings significant health benefits are usually obtained within 6 months of starting ART; mortality can be reduced by up to 78% [6], rates of opportunistic infections reduced by 56%[33], and robust immunological gains obtained[28]. We saw good survival and immunological outcomes at 6 months. People also became informed about their illness and the benefits of treatment. This improved health and knowledge may enable them to better manage their illness if treatment stops, reduce the risk of them transmitting the virus by adapting behaviour, and help them to potentially seek treatment elsewhere as it becomes increasingly available in resource-limited settings [19].

Predictions of how long people will be able to take treatment and what will happen in the future are rarely possible. Where there is uncertainty people should be given the chance of receiving treatment, but informed patient consent should be obtained regarding risks, benefits, and potential for interruption or cessation of ART. However it is important to determine the minimum time that treatment should be available to obtain benefit. While absolute rules on this are difficult, we and others feel that around 3-6 months on treatment should be seen as a minimum [15].

### 4. "Stopping treatment will lead to resistance."

It is clear that while regular treatment interruptions do promote resistance, the risk of developing resistance due to a single stop of treatment is low. The risk is further reduced if those taking an NNRTI-based regimen receive a 1-week continuation of dual nucleoside reverse transcriptase inhibitor (NRTI) therapy (ie AZT/3TC or D4T/3TC; a 'washout' course) to cover the drug's longer half-life [34], or, for those on a PI-based regimen, all drugs are stopped together [35].

### 5. "Resources should be directed to other more important acute health priorities."

In high HIV prevalence areas many competing health priorities such as malaria, diarrhoea, and tuberculosis occur more frequently and have a higher mortality due to the presence of underlying HIV. Addressing underlying HIV substantially contributes to addressing these needs and reduces their demands on medical services. In our experience, integration of HIV activities strengthened other health activities such as the diagnosis and treatment of tuberculosis and maternal and reproductive health by sharing resources and improving laboratory services, procurement, supply, and monitoring mechanisms. This contributes to improving the health of all victims of conflict but also increases the likelihood of sustainability for implemented activities [see Additional file 1, Table S1] [12].

Nevertheless, there are certainly low HIV-prevalence settings or especially difficult and unstable environments where the potential benefits of introducing ART may not justify the resources required. In some conflict-affected areas such as Somalia, Darfur, and Sri Lanka, MSF-OCA has not introduced HIV treatment activities (apart from post-exposure prophylaxis) because of a lack of HIV-related medical needs identified by field teams, often compounded by potentially serious negative consequences for those testing positive. A decision to provide treatment should, like all health-care decisions, be made on an informed and unprejudiced assessment of the needs and priorities of the population and the feasibility of an effective response.

### 6. "HIV programmes should be sustainable"

Continuation or handover of HIV programmes started in a conflict-affected area, usually without prior HIV activities, can pose significant challenges. Our experience was that an established and effectively running programme was a catalyst to engage other actors, especially National Aids Programmes and MoHs, to provide ART in the region [12]. Despite the difficult conditions in which the programmes were instituted, a handover partner was found for all programmes that MSF-OCA closed [see Additional file 1, Table S1]. All programmes were handed over to MoH, sometimes with national or international non-governmental organisations providing technical support, training, support to procurement and supply channels, and funding of key staff. For example, in Mindouli, Republic of Congo, the National AIDS Programme funded key staff involved in HIV activities and accredited the hospital as an ART site [12]. Early integration with existing MoH systems and structures and planning and discussion with potential partners facilitated the process. Identification and onsite training of key staff to remain in the programme was vital.

### Further challenges in conflict environments

#### Prevention of Mother to Child Transmission (PMTCT)

Despite the recognised importance of the intervention and a strong desire to implement it by programme managers, the initial inclusion of PMTCT was surprising difficult in many settings, usually due to the resistance of health-care staff. Their concerns included perceived potential negative consequences for women diagnosed HIV-positive, the complexity of the intervention in environments where programme disruption was possible, a lack of understanding of its potential benefits, and limited staff experience in managing HIV. Nevertheless, HIV transmission rates can be reduced by partial interventions even if full ones are prevented by programme disruption [36]. Our experience was that with simplified protocols and tools, quality education and counselling of women and staff, and the provision of extra resources, PMTCT activities were possible. Infant feeding was complicated by the potential for programme interruption to leave mothers who formula fed without infant feeding options, and therefore exclusive breastfeeding with early rapid weaning at 6-9 months was usually promoted, apart from Angola where almost all women were offered and adopted formula feeding.

### Paediatric HIV care

Similarly to programmes in stable settings, the inclusion of infants and children was limited. This was influenced by difficulties diagnosing HIV in children, low clinician confidence in clinical HIV paediatric care, and lack of drugs in adapted formulations and fixed-dose combinations [37]. Strategies targeting children, especially orphans, and improved and adapted diagnostic tools and medications are needed in these settings.

## Conclusion

The primary benefit of introducing ART in conflict-affected settings is the reduction of HIV-related morbidity and mortality in affected populations, giving hope and health to people slowly dying in environments characterized by loss, displacement, violence, and trauma. Additional benefits include strengthening of health systems and increased morale of health staff and the community when able to treat 'dying' patients and tackle HIV/AIDS. In our experience this acts strongly to reduce HIV-related stigma and discrimination further encouraging people to seek HIV testing and care, allows prevention strategies to be more openly discussed and implemented, and vitally improves the level of support for HIV-infected people by family, health staff, and the community. It also leads to increased HIV awareness and knowledge in the population, and in many programmes to the creation of community support and advocacy groups which can strengthen the community's response to HIV and AIDS. Furthermore, the introduction of specific activities such as ART, post-exposure prophylaxis, and PMTCT can have a significant impact on HIV transmission, and thus may counteract the risks of an increasing HIV epidemic in these areas, especially in the post-conflict period.

Our experience in providing medical care to populations in conflict-affected areas, especially in sub-Saharan Africa, shows that in many of these settings there are significant HIV-related urgent health needs. This study has reaffirmed previous reports that with commitment, simplified treatment and monitoring, programmatic adaptations for the conditions, and resources, HIV treatment including ART can be feasibly and effectively provided in conflict or post-conflict settings with many secondary benefits. We hope our experience will encourage and help others to include HIV treatment in their health interventions where conflict and HIV-related health needs overlap.

## Appendix 1: Practical measures for safe and effective HIV care in conflict settings

### 1. Design programmes to be resilient to disruption

• Simplify management and treatment protocols as much as possible

• Human resources planning: 1) a dedicated member of staff should be in charge of instability preparedness and training; 2) develop multiskilled staff and community group involvement to enable coverage if medical staff are evacuated

• Halt initiation of patients on ART if acute instability occurs or is imminent. Non-essential services (eg general HIV education and testing) can be minimized and the frequency of follow-up and monitoring reduced

• Alternative site(s) for care delivery should be identified with community consultation taking into account routes likely to be used by the fleeing population.

• Alternative sources of care - identify programmes in neighbouring regions/countries that might be accessed by patients if migration occurred related to instability. Reciprocal arrangements for care should be agreed, and patients should carry treatment "passports" with information such as clinical illnesses, current HIV drug regimens, adverse reactions, and relevant laboratory results.

• Communication networks (radio, mobile phones, and church and community groups) with staff and patients are essential during periods of instability.

• Inform all parties to the conflict and community leaders about the importance of maintaining the project for their people, and ensure a clear image of independence and neutrality for health care

### 2. Focus on adherence

• Patients must be educated and motivated to adhere to medications in the event of disruption. They should be advised not to conserve drugs, to take their drugs even if they have no food, and not to share medications or obtain drugs from sources where quality cannot be assured. Partners or a 'treatment buddy' can ensure adherence support if staff are evacuated.

### 3. Emergency drug stocks and forced treatment interruption

• Patients should have enough ARV drugs to cover a short disruption of drug delivery. We recommend a 'runaway stock' of 1-3 months of treatment (including prophylaxis, especially cotrimoxazole) kept by the patient or given when disruption is anticipated. Patients should bring this to the clinic on each visit so that stock and expiry can be checked.

• If TB treatment has commenced, patients should be allotted enough medication to complete a full course; this should be stored in the clinic and given to the patient if instability is predicted. Patients should be educated on how to take their medications, with many of the same adherence rules in times of crisis as for ARVs plus information on managing a safe treatment interruption by ceasing all TB drugs at once if unavoidable.

• Patients should be given clear information regarding conditions under which they should stop treatment and how to reduce risks associated with disruption. If a washout course (dual NRTI tail for 1 week) is required it should ideally be given immediately prior to instability, but can be given at the start of treatment in contexts where instability is likely, to be kept and taken if treatment disruption occurs.

### 4. Security of drug stocks

• Large amounts of drugs are a risk for looting. Stock should be locked securely in a discreet location. A buffer stock should be available, but it might be necessary to have excess stock evacuated if looting is a risk. Large stocks should not be kept in high-risk sites. Dividing stock and storing it at different locations minimises the risk of losing all of it or of all stock being inaccessible for security reasons.

## Consent

Written informed consent was obtained from the patient for publication of this report and accompanying images. A copy of the written consent is available for review by the Editor-in-Chief of this journal.

## Competing interests

The authors declare that they have no competing interests.

## Authors' contributions

DO'B conceived and wrote and researched the paper. SV helped design, write, and research the paper. JG did statistical analyses. LS helped with research and writing of the paper. TE helped with research and writing of the paper. KS helped with research and writing of the paper. LF helped with research and writing of the paper. CM helped with design, research and writing of the paper. All authors read and approved the final manuscript.

## Supplementary Material

Additional file 1 Table S1: Programme summaries and context
Programme contexts and settings. Click here for file

Additional file 2 **Table S2: Project data on HIV prevalence and patient numbers**. Programme data and outcomes. Table S3: Baseline and outcome information on adult patients commenced on ART. Programme data and outcomes. Click here for file
