# Supplementary material for: Provision of antiretroviral treatment in conflict settings: the experience of Médecins Sans Frontières
Source: Confl Health. 2010 Jun 17;4:12. doi: 10.1186/1752-1505-4-12 (PMC2911421; doi:10.1186/1752-1505-4-12)
Supplement: Additional file 1 — Table S1: Programme summaries and context Programme contexts and settings. [file 1752-1505-4-12-S1.DOC]

Table S1: Programme summaries and context

| **Project (Country)** | **Period of active conflict [[1]](#footnote-2)** | **Dates of MSF ART activities** | **Conflict/ post conflict at time of starting HIV activities** | **Type of MSF programme** | **Context** | **Estimated national HIV prevalence[[2]](#footnote-3),[[3]](#footnote-4)** | **Estimated national ART coverage rates[[4]](#footnote-5)** | **HIV activities introduced** | **Lab services*** | **Closure or handover of ART activities** |
| --- | --- | --- | --- | --- | --- | --- | --- | --- | --- | --- |
| **Malange (Angola)** | 1975-2002 | September 2004 - June 2007 | Post-conflict | Vertical: district hospital and health centres | Urban | 2% | 6% | VCT, OI, ART, HIV/TB, PMTCT | CD4, ALT, Xray | Handed over to MoH supported by Brazilian Corporation |
| **Kayanza (Burundi)** | 1993-2006 | Not applicable – refer to MoH in district hospital | Post conflict | Integrated BHC: regional hospital and health centres. | Rural | 3-4% | 14% | VCT, OI, HIV/TB |  | Handover to MoH supported by EU |
| **Kinyinya (Burundi)** |  | November 2005 –September 2007 |  | Integrated BHC: regional hospital and health centres. |  |  |  | VCT, OI, ART, HIV/TB, PMTCT | ALT, Xray | Handover to MoH supported by EU |
| **Ruyigi (Burundi)** |  | November 2005 - June 2006 |  | Integrated BHC: district hospital and health centres. |  |  |  | VCT, OI, ART, HIV/TB | ALT, Xray | Handover to MoH |
| **Bougila (CAR)** | Frequent instability since 1960; armed rebellion from 2005-present | January 2007 - | Conflict | Integrated BHC: regional hospital and health centres | Rural | 6-7% | 3% | VCT, OI, ART, HIV/TB, PMTCT | CD4, ALT | Ongoing |
| **Sincelejo (Colombia)** | 1964/66-present | Not applicable – refer to MoH in district hospital | Conflict | Integrated with focus on reproductive and mental health: health centres | Urban | 1-2% | 43% | VCT, OI | CD4, ALT, Xray | Ongoing |
| **Danane (Cote d’Ivoire)** | 2002-2007 | October 2005 – March 2008 | Conflict | Integrated BHC: district hospital and health centres | Rural | 5-10% | 17% | VCT, OI, ART,  HIV/TB, PMTCT | CD4, ALT, Xray, VL | Handover to MoH supported by Elizabeth Glazer foundation |
| **Dubie (DRC-Katanga)** | 1998-2006 | August 2007 - | Post-conflict | Integrated BHC: district hospital and health centres | Rural | 2-5% | 4% | VCT, OI, ART, HIV/TB, PMTCT | ALT | Ongoing |
| **Kilwa (DRC-Katanga)** |  | June 2007 - |  | Integrated BHC: regional hospital and health centres |  |  |  | VCT, OI, ART, HIV/TB, PMTCT | ALT, Xray | Ongoing |
| **Baraka (DRC- Kivus)** | 1998 to present | October 2006 - | Conflict | Integrated BHC: regional hospital plus health centres | Rural |  |  | VCT, OI, ART, HIV/TB |  | Ongoing |
| **Bukavu (DRC- Kivus)** |  | October 2003 – January 2009 |  | Vertical: district and regional hospitals and health centres | Urban |  |  | VCT, OI, ART, TB, PMTCT | CD4, ALT, Xray, VL | Handover to MoH supported by GTZ |
| **Shabunda (DRC- Kivus)** |  | June 2006 - |  | Integrated BHC: regional hospital plus health centres | Rural |  |  | VCT, OI, ART, HIV/TB, PMTCT |  | Ongoing |
| **Walikale (DRC- Kivus)** |  | June 2006 – September 2008 |  | Integrated BHC: regional hospital plus health centres |  |  |  | VCT, OI, ART, HIV/TB |  | Handover to MoH |
| **Manipur (India)** | 1950s to present | January 2006 - | Conflict | Parallel integrated BHC: health centres | Rural | 1-2% | 7% | VCT, OI, ART, HIV/TB, PMTCT | CD4, ALT, Xray, VL | Ongoing |
| **Nimba (Liberia)** | 1989-2003 | November 2005 - April 2007 | Post-conflict | Integrated BHC: district hospital and health centres | Rural | 2-5% | 3% | VCT, OI, ART, HIV/TB, PMTCT | CD4, ALT | Handover to MoH supported by national NGO Africare |
| **Kindamba (RoC)** | 1993-95; 1997-99; 2002-03 | September 2006 –May 2008 | Conflict | Integrated BHC: regional hospital and health centres | Rural | 3-8% | 17% | VCT, OI, ART, HIV/TB | CD4, ALT | Transfer to MoH programme in Mindouli |
| **Kinkala (RoC)** |  | May 2005 - May 2006 |  | Integrated BHC: district hospital and health centres |  |  |  | VCT, OI, ART,  HIV/TB | CD4, ALT, Xray | Transfer to MSF programme in Mindouli |
| **Mindouli (RoC)** |  | March 2006 – February 2008 |  | Integrated BHC: regional hospital and health centres |  |  |  | VCT, OI, ART, HIV/TB | CD4, ALT | Handover to MoH |
| **Kambia (Sierra Leone)** | 1991-2002 | December 2004 –August 2006 | Post conflict | Integrated BHC: hospital and health centres | Rural | 1-2% | 2% | VCT, OI, ART, PMTCT | CD4, ALT, Xray | Handover to MoH |
| **Magburaka (Sierra Leone)** |  | January 2005 – August 2006 |  | Integrated BHC: hospital and health centres |  |  |  | VCT, OI, ART, PMTCT | CD4, ALT, Xray | Handover to MoH |
| **Malakal**  **(southern Sudan)** | 1983-2005 | December 2005 -July 2006 | Post conflict | Integrated BHC: regional hospital and health centres | Rural | 2.5% | 1%[[5]](#footnote-6) | VCT, OI, ART, HIV/TB |  | Handover to MoH |
| **Nasir (southern Sudan)** |  | June 2007 - |  | Integrated BHC: district hospital and health centres |  |  |  | VCT, OI, ART, HIV/TB, PMTCT |  | Ongoing |
| **Lira (Uganda)** | 1986-present | September 2006 -April 2007 | Conflict | Integrated BHC: health centres and nutrition programme | Rural, refugee camp | 6-8% | 51% | OI, ART, HIV/TB | Xray | Handover to MoH |
| **Kitgum (Uganda)** |  | February 2007 - |  | Integrated BHC: health centres | Rural, Refugee camp |  |  | VCT, OI, ART, HIV/TB, PMTCT | CD4, ALT, Xray | Ongoing |

- All programmes had access to Haemoglobin testing and Ziehl-Neelson staining for mycobacteria
- RoC= Republic of Congo, DRC= Democratic Republic of Congo, CAR= Central African Republic, BHC= basic health care, VCT= voluntary counseling and testing, OI= opportunistic infection, ART= antiretroviral therapy, PMTCT= prevention of mother to child transmission, TB= tuberculosis, ALT= alanine transaminase, VL= viral load, GTZ= Deutsche Gesellschaft für Technische Zusammenarbeit

1. Dates for ends of conflict are ceasefire/peace agreements but often instability and regional violence persisted for some time, in particular in RoC (where a peace agreement was not signed with the local warlord until 2007) and DRC. Sources vary with respect to some dates of some conflicts. Data are from MSF records, Reuters crisis briefings <http://www.alertnet.org/db/crisisprofiles>, Human Rights Watch <http://www.hrw.org/reports/2008/india0908/2.htm>, BBC country profiles <http://news.bbc.co.uk/1/hi/world/africa/country_profiles/1076836.stm> , and UNHCR <http://www.unhcr.org/cgi-bin/texis/vtx/page?page=49e45c156> (all accessed August 2009) [↑](#footnote-ref-2)
2. 2005 figures from reference 18. [↑](#footnote-ref-3)
3. This may not reflect the true HIV prevalence in the areas where MSF was working [↑](#footnote-ref-4)
4. 2005 figures from reference 19. Note that this might not reflect the ART coverage in the areas where MSF was working. [↑](#footnote-ref-5)
5. Figure is for all of Sudan not just southern Sudan [↑](#footnote-ref-6)
