# Supplementary material for: Provision of antiretroviral treatment in conflict settings: the experience of Médecins Sans Frontières
Source: Confl Health. 2010 Jun 17;4:12. doi: 10.1186/1752-1505-4-12 (PMC2911421; doi:10.1186/1752-1505-4-12)
Supplement: Additional file 2 — Table S2: Project data on HIV prevalence and patient numbers. Programme data and outcomes. Table S3: Baseline and outcome information on adult patients commenced on ART. Programme data and outcomes. [file 1752-1505-4-12-S2.DOC]

Table S2: Project data on HIV prevalence and patient numbers

| **Country** | **Project** | **HIV prevalence overall** | **HIV prevalence in TB patients** | **HIV prevalence in ANC patients** | **Comments on testing profile** | **Number of HIV+ patients registered under care** | **Cumulative number of patients started on ART *(adult;child)*** | **At analysis (adults)*** | | | |
| --- | --- | --- | --- | --- | --- | --- | --- | --- | --- | --- | --- |
|  |  |  |  |  |  |  |  | **Still on ART (%)** | **Dead**  **(%)** | **LFU**  **(2 months)**  **(%)** | **Transfer**  **(%)** |
| **Angola** | **Malange** | 2% | 10% | 1% | Focus on medical inpatients, TB, ANC | 615 | 159 ; 7 | 111  (73%) | 19  (12%) | 29  (18%) | 0 |
| **Burundi** | **Kayanza** | 12% | 12% | Not applicable | Focus on medical inpatients and TB | 40 | Not applicable |  |  |  |  |
|  | **Kinyinya** | 5% | 11% | 4% | Included community VCT, medical inpatients and TB | 123 | 33 ; 1 | 31  (94%) | 2  (6%) | 0  (0%) | 0 |
|  | **Ruyigi** | 4% | No data | Not applicable | Included community VCT, medical inpatients and TB | 54 | 5 ; 0 (referred MoH) | No data | No data | No data | No data |
| **CAR** | **Boguila** | 35% | 45% | 1% | Focus on medical inpatients, TB, ANC | 273 | 116 ; 4 | 92  (79%) | 7  (6%) | 7  (6%) | 2  (2%) |
| **Colombia** | **Sincelejo** | 12% | No data | Not applicable | Focus on medically unwell patients | No data | Not applicable |  |  |  |  |
| **Cote d’Ivoire** | **Danane** | 14% | 33% | 3% | Focus on medical inpatients, TB, ANC | 1826 | 613 ; 43 | 396  (68%) | 50  (8%) | 98  (17%) | 18  (3%) |
| **DRC (Katanga)** | **Dubie** | 19% | 8% | Not applicable | Focus on medical inpatients and TB | 144 | 52 ; 3 | 35  (67%) | 11  (21%) | 5  (10%) | 0 |
|  | **Kilwa** | 22% | 17% | Not applicable | Focus on medical inpatients and TB | 238 | 40 ; 1 | 30  (75%) | 9  (23%) | 1  (2%) | 0 |
| **DRC (Kivus)** | **Baraka** | 14% | 13% | Not applicable | Focus on medical inpatients and TB | 286 | 94 ; 7 | 54  (57%) | 18  (19%) | 18  (19%) | 4  (4%) |
|  | **Bukavu** | 10% | 5% | 2% | Community VCT, ANC, STI, TB, hospitalised patients | 3412 | 1577 ; 137 | 1037  (68%) | 197  (13%) | 188  (12%) | 87  (6%) |
|  | **Shabunda** | 6% | 7% | 1% | Focus on medical inpatients, TB, ANC | 63 | 57 ; 6 | No data | No data | No data | No data |
|  | **Walikale** | 9% | 9% | No data | Focus on medical inpatients, TB, ANC | 58 | 29 ; 0 | 16  (55%) | 1  (3%) | 0 | 7  (24%) |
| **India** | **Manipur** | 14% | 60% | 2% | High proportion of IVDU | 1657 | 821 ; 76 | 360  (44%) | 51  (6%) | 85  (10%) | 282  (34%) |
| **Liberia** | **Nimba** | 4% | 12% | 2% | Focus on medical inpatients, TB, ANC | 206 | 59 ; 0 | 55  (93%) | 2  (4%) | 2  (4%) | 0 |
| **RoC** | **Kindamba** | 41% | 19% | Not applicable | Focus on medical inpatients and TB | 40 | 23 ; 1. | 15  (65%) | 3  (13%) | 0 | 4  (17%) |
|  | **Kinkala** | 28% | No data | Not applicable | Focus on medical inpatients and TB | 79 | 52 ; 4 | 42  (82%) | 7  (14%) | 3  (6%) | 0 |
|  | **Mindouli** | 37% | 34% | Not applicable | Focus on medical inpatients and TB | 357 | 222 ; 12 | 171  (71%) | 18  (8%) | 23  (10%) | 10  (4%) |
| **Sierra Leone** | **Kambia** | 6% | No data | 2% | Focus on medical inpatients and ANC | 218[[1]](#footnote-2) | 39 ; 2 | 29  (74%) | 8  (21%) | 2  (5%) | 0 |
|  | **Magburaka** | 2% | No data | 1% | Focus on medical inpatients and ANC | No data | 62 ; 0 | 46  (74%) | No data | No data | No data |
| **South Sudan** | **Malakal** | 8% | 4% | Not applicable | Focus on TB and KA | 26 | 8 ; 0 | 4  (50%) | 3  (38%) | 0 | 1  (13%) |
|  | **Nasir** | 5% | 6% | Not applicable | Focus on TB, STI, medical inpatients and KA | 47 | 12 ; 0 | 2  (17%) | 6  (50%) | 4  (33%) | 0 |
| **Uganda** | **Kitgum** | 12% | 78% | 5% | Focus on TFC children and their parents | 459 | 118 ; 10 | 105  (90%) | 9  (8%) | 0 | 2  (2%) |
|  | **Lira** | 45% | 72% | Not applicable | Focus on TFC children and their parents | 294 | 16 ; 34 | 14  (88%) | 1  (6%) | 1  (6%) | 0 |
| **ALL PROJECTS** | ***Total***  ***(Median)*** | (12%) | (13%) | (2%) |  | 10515  (212) | 4207 ; 348  (55 ; 16) | 2645  (64%) | 422  (10%) | 466  (11%) | 417  (10%) |

* Data not available for Ruyigi, Burundi and Shabunda, DRC

Table S3: Baseline and Outcome information on adult patients commenced on ART *

| **Project** | **Country** | **ART Baseline data** | | | | | | **At analysis** | **After 6 months of ART** | | | **After 12 months ART** | |
| --- | --- | --- | --- | --- | --- | --- | --- | --- | --- | --- | --- | --- | --- |
|  |  | **Age: median years [IQR]** | **Gender: female n (%)** | **BMI: median kg/m2 [IQR]** | **Prior ARV experience: naïve n (%)** | **WHO stage: stage 3/4 n (%)** | **CD4: median cells/mm3 [IQR]** | **Months on ART: median [IQR]** | **CD4 gain: median [IQR]** | **survival**  **(95% CI)** | **% dead / % LFU**  **(n patient)** | **survival**  **(95% CI)** | **% dead / % LFU**  **(n patient)** |
| **CAR** | **Boguila** | 30.4  [26.2, 36.1] | 57  (67.9%) | 18.8  [16.6, 21.2] | 82 (96.5%) | 75 (88.2%) | 147  [65, 230] | 5.4  [0.7, 12.7] | Not done | 0.94  (0.84, 0.98) | 6% / 6%  (67) | 0.94  (0.80, 0.99) | 5% / 3%  (39) |
| **Cote d’Ivoire** | **Danane** | 33.1  [27.0, 40.1] | 397 (74.8%) | 18.4  [16.4, 20.8] | 521  (97.4%) | 401 (75.0%) | 159  [66, 233] | 7.0  [2.0, 14.5] | 184  [60, 279] | 0.93  (0.90, 0.95) | 6% / 17%  (480) | 0.90  (0.86, 0.93) | 9% / 18%  (327) |
| **DRC – Katanga** | **Dubie** | 36.3  [32.0, 42.1] | 25 (65.8%) | 16.2  [14.3, 17.7] | 37 (97.4%) | 37 (97.4%) | 235  [120, 350] | 2.7  [0.9, 8.6] | Not done | 0.70  (0.49, 0.84) | 28% / 14%  (29) | 0.70  (0.39, 0.88) | 27% / 7%  (15) |
|  | **Kilwa** | 34.9  [30.0, 42.3] | 25 (62.5%) | 18.3  [16.5, 20.3] | 34 (85.0%) | 37 (92.5%) | Not done | 13.3  [8.3, 18.5] | Not done | 0.77  (0.61, 0.87) | 22% / 2%  (40) | 0.73  (0.54, 0.85) | 26% / 3%  (34) |
| **DRC - Kivus** | **Baraka** | 37.2 [30.0, 45.9] | 59 (74.7%) | 19.7  [17.3, 21.2] | 66 (83.5%) | 74 (93.7%) | Not done | 6.2  [2.2, 12.1] | Not done | 0.82  (0.72, 0.90) | 16% / 15%  (73) | 0.79  (0.63, 0.88) | 18% / 24%  (51) |
|  | **Bukavu** | 37.4  [30.8, 43.9] | 1069 (68.7%) | 20.0  [18.2, 22.0] | 1506 (96.8%) | 1215 (91.9%) | 126  [51, 211] | 17.6  [5.4, 33.6] | 149  [82, 222] | 0.92  (0.90, 0.93) | 8% / 6%  (1443) | 0.89  (0.87, 0.90) | 11% / 10%  (1289) |
|  | **Walikale** | 38.9  [32.0, 42.0] | 17 (60.7%) | 20.1  [17.7, 21.6] | 20 (69.0%) | 28 (96.6%) | Not done | 9.4  [6.3, 15.2] | Not done | 1.00  () | 0% / 0%  (25) | 1.00  () | 0% / 0%  (13) |
| **India** | **Manipur** | 33.7  [29.4, 38.1] | 344 (48.2%) | 20.0  [18.3, 21.9] | 642 (90.0%) | 372 (52.2%) | 145  [84,191] | 14.6  [7.1, 24.4] | 106  [46, 168] | 0.96  (0.95, 0.97) | 4% / 5%  (745) | 0.95  (0.93, 0.97) | 4% / 9%  (639) |
| **RoC** | **Kindamba** | 41.0  [31.3, 46.7] | 17 (73.9%) | 16.6  [14.8, 18.5] | 11 (47.8%) | 23 (100%) | 70 [27,162] | 6.7  [3.0, 15.0] | 347  [163, 498] | 0.82  (0.54, 0.94) | 18% / 0%  (17) | 1.00  () | 0% / 0%  (9) |
|  | **Kinkala** | 35.2  [30.0, 38.7] | 36 (70.6%) | 17.9  [15.9, 20.4] | 49 (96.1%) | 46 (90.2%) | 76  [27,136] | 3.6  [0.9, 6.3] | Not done | 0.81  (0.60, 0.92) | 16% / 6%  (32) | 1.00  () | 0% / 0%  (3) |
|  | **Mindouli** | 37.0  [32.0, 43.0] | 153 (68.9%) | 17.9  [16.4, 19.4] | 211 (95.0%) | 204  (91.9%) | 104  [40, 172] | 7.6  [3.9, 14.9] | 89 [48, 122] | 0.94  (0.89, 0.96) | 6% / 5%  (190) | 0.90  (0.83, 0.95) | 8% / 13%  (124) |
| **Uganda** | **Kitgum** | 36.1  [30.1, 43.1] | 55 (59.1%) | 17.8  [16.2, 19.6] | 90 (96.8%) | 63 (67.7%) | 154.5  [100, 186] | 6.6  [1.4, 10.6] | 141 [100, 180] | 0.92  (0.83, 0.96) | 8% / 3%  (78) | 0.85  (0.66, 0.94) | 14% / 0%  (29) |
| **ALL PROJECTS** | ***Total***  ***(Median)*** | 35.2  [29.4, 41.4] | 66% | 19.5  [17.4, 21.6] | 94% | 80% | 139  [63, 211] | 11.8  [3.9, 22.7] | 129  [63.75, 197] | 0.92  (0.91, 0.93) | 7% / 8%  (3219) | 0.89  (0.88, 0.91) | 9% / 11%  (2572) |
| **ART-LINC projects ^29^** | ***(Median)*** | (36) | (51%) |  |  | (67%)** | (108) |  | (106) |  |  |  | (6% / 15%) |

*Analysis limited to 12 programmes due to either loss of Fuchia data post-closure (2 projects - Kininya and Nimba) or because Fuchia was not implemented (8 projects)..

** Of those with known WHO stage.

RoC = Republic of Congo, DRC = Democratic Republic of Congo, CAR = Central African Republic, ANC = antenatal care, VCT = voluntary counselling and testing, ART = antiretroviral therapy, TB = tuberculosis, MoH = Ministry of Health, LFU = lost to follow-up, STI = sexually-transmitted infection, IVDU = intravenous drug users, KA = kala azar, TFC = therapeutic feeding centre.

1. Many of these entered prevention of mother to child transmission (PMTCT) [↑](#footnote-ref-2)
